# Proximal Myopathy With Hypercalcemic Crisis and Papillary Thyroid Carcinoma in a 38-Year-Old Female

**DOI:** 10.7759/cureus.20700

**Published:** 2021-12-26

**Authors:** Muhammad Rezeul Huq, Ahmed Hossain, M. A. Hannan, Mahin Binte Anwar, Ahad Mahmud Khan

**Affiliations:** 1 Department of Neurology, Combined Military Hospital, Dhaka, BGD; 2 Department of Medicine, Police Hospital, Gazipur, BGD; 3 Department of Neurology, Bangabandhu Sheikh Mujib Medical University, Dhaka, BGD; 4 Department of Radiology, Combined Military Hospital, Dhaka, BGD; 5 Epidemiology, Projahnmo Research Foundation, Dhaka, BGD; 6 Usher Institute, The University of Edinburgh, Edinburgh, GBR

**Keywords:** hypercalcemic crisis, papillary thyroid carcinoma, parathyroid crisis, primary hyperparathyroidism, proximal myopathy

## Abstract

Although proximal myopathy is a well-known manifestation of primary hyperparathyroidism (PHP), it is usually not the first one. Here, we present the case of a 38-year-old female who presented to the neurology outpatient department with proximal myopathy as the presenting feature of PHP along with a hypercalcemic crisis. Her serum calcium and intact parathyroid hormone levels were very high. Her symptoms and calcium levels improved with adequate hydration and bisphosphonate therapy. Ultrasonography of the thyroid and parathyroid glands and Tc99m sestamibi single-photon emission computed tomography-computed tomography of the parathyroid glands suggested adenoma or carcinoma of the parathyroid gland on the right side with another irregular right-sided thyroid nodule. Electromyography showed low-amplitude polyphasic potentials suggestive of myopathy. Subsequently, the patient underwent surgical resection of both the right parathyroid glands and the thyroid nodule. Histopathology report was suggestive of parathyroid adenoma and papillary thyroid carcinoma. Hypercalcemic crisis is a rare clinical scenario, which needs prompt diagnosis and treatment. Otherwise, the condition may have a fatal outcome. Due to its diverse presentation, physicians should be aware of this condition. Moreover, we need to be cautious in treating a patient having hyperparathyroidism with thyroid nodule due to possible concomitant thyroid malignancy.

## Introduction

Primary hyperparathyroidism (PHP) is most commonly diagnosed incidentally during the evaluation of hypercalcemia. In symptomatic cases, patients may have variable presentations such as renal stone, psychiatric manifestations, lethargy, increased thirst, abdominal pain, peptic ulceration, hypertension, and bone disease. The typical presentation of PHP has evolved over the last few decades. Classical renal, neuromuscular, or musculoskeletal presentations are rarely seen nowadays. A PHP patient can present with various neuromuscular symptoms; however, an initial presentation with florid proximal myopathy is almost unheard of. The hypercalcemic crisis or parathyroid storm or parathyroid crisis, which is a life-threatening medical emergency, is also considered an unusual presentation of PHP [1].

To the best of our knowledge, this is the first report of PHP presenting with both proximal myopathy and hypercalcemic crisis. Although papillary thyroid carcinoma (PTC) is a common thyroid malignancy, its association with PHP is uncommon. Few cases of PTC have been found during the evaluation of PHP [2]. Here, we describe a case of PHP associated with PTC presenting with rare presentations of proximal myopathy and hypercalcemic crisis.

## Case presentation

A 38-year-old female presented to the neurology outpatient department (OPD) of Bangabandhu Sheikh Mujib Medical University (BSMMU) in October 2019 complaining of weakness and wasting of all four limbs for three months. Her weakness was progressive and more marked proximally, and she could not walk without assistance. Other than weakness, she did not have any other complaints. The patient was admitted to the neurology ward. After admission, she developed repeated vomiting, increased thirst, frequent urination, and recurrent colicky upper abdominal pain. Apart from anxiety, she had no other psychiatric complaints. The patient did not have any family history of endocrine or neoplastic diseases. She was alert, oriented, and calm, and her vitals were normal. A small non-tender round nodule measuring approximately 2 × 1.5 cm was noted over the right thyroid lobe. She had wasting of proximal parts of all four limbs, more marked in lower limbs, especially the quadriceps. No fasciculations were noted. Muscle power was grade 3 proximally and grade 5 distally in all four limbs. All jerks were exaggerated, and the plantar reflex was flexor on both sides. Gait was waddling type, and Gower’s sign was positive.

**Figure 1:**
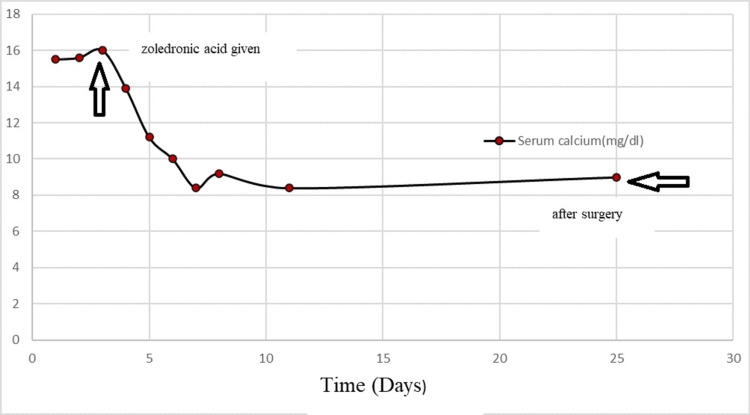
Variation of calcium levels (mg/dL) over time (days).

**Figure 2:**
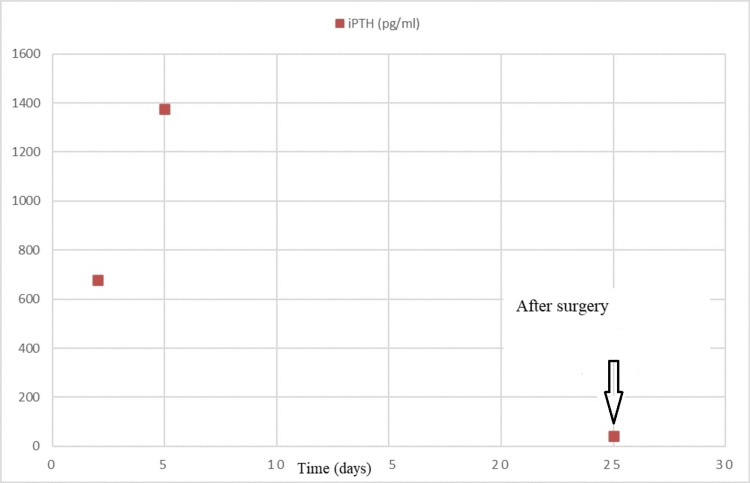
Variation of iPTH levels over time (days). iPTH: intact parathyroid hormone

**Figure 3:**
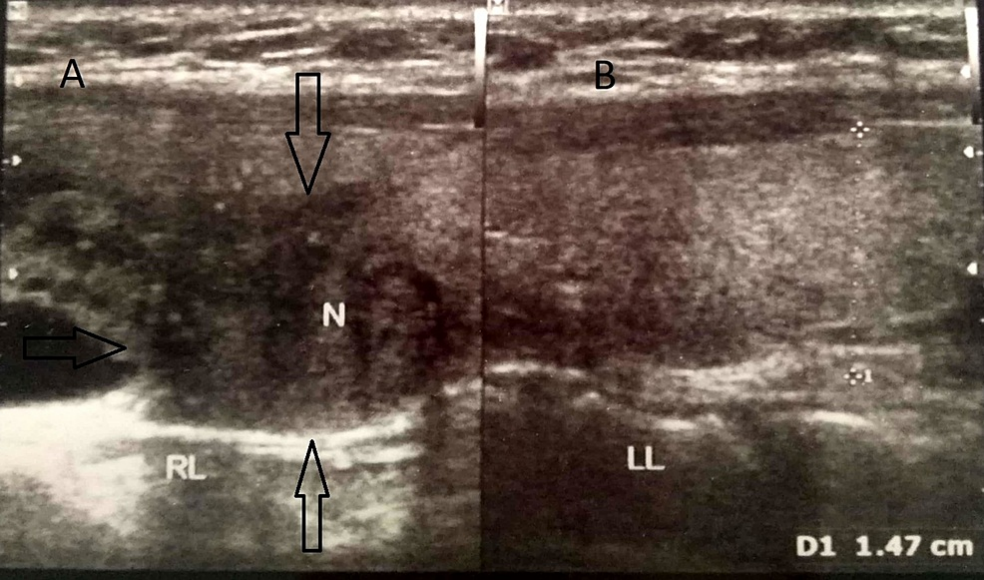
(A) USG of the thyroid and parathyroid glands showing (arrows) an irregular hypoechoic nodular lesion measuring approximately 17 × 14 mm having foci of calcification in the upper pole of the right lobe of the thyroid gland suggestive of adenoma or carcinoma of the parathyroid gland. (B) No abnormalities were found on the left side. USG: ultrasonography

**Figure 4:**
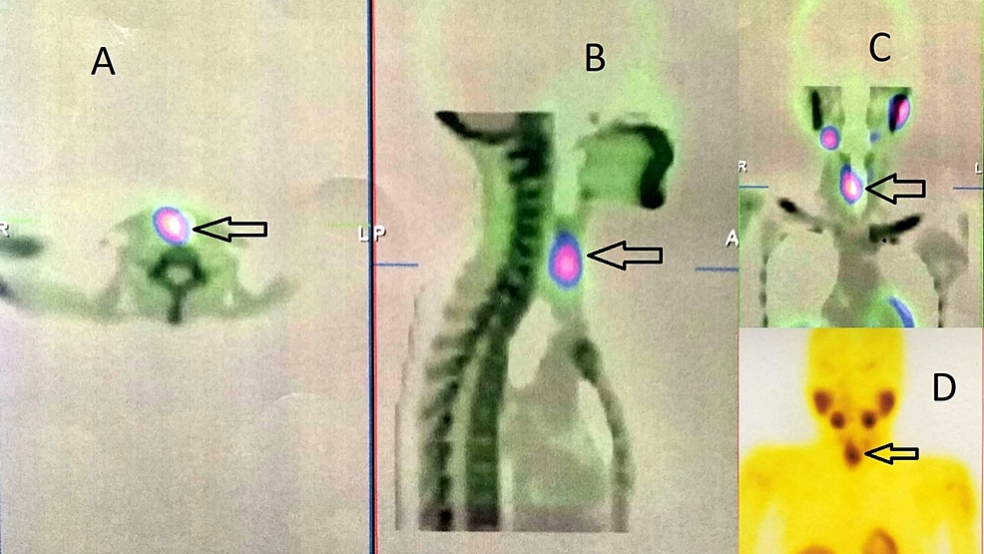
Transaxial (A), sagittal (B), and coronal (C) SPECT-CT of parathyroid glands showing (arrows) focal area of increased radiotracer concentration in the right side of the neck at the level of C5-C7 vertebra suggestive of parathyroid adenoma or carcinoma. Delayed static image of Tc99m sestamibi parathyroid scintigraphy (D) showing (arrow) persistent focal activity in the abovementioned area. SPECT-CT: single-photon emission computed tomography-computed tomography

**Figure 5:**
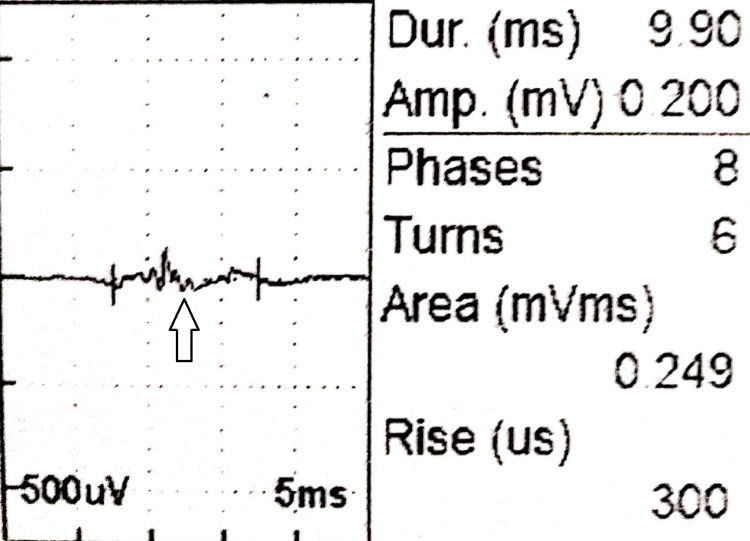
EMG tracing showing small-amplitude polyphasic myopathic potential (arrow). EMG: electromyography

On admission, the patient’s corrected serum calcium, intact parathyroid hormone (iPTH), and inorganic phosphate levels were 16 mg/dL (8.5-10.5 mg/dL), 677.3 pg/mL (7-53 pg/mL), and 2.3 mg/dL (2.3-4.7 mg/dL), respectively (Figures 1, 2). She was treated with adequate hydration (both oral and intravenous fluids), injectable proton pump inhibitors, and intravenous zoledronic acid. The patient was under the close supervision of a team comprising neurologists, endocrinologists, and cardiologists. Her electrocardiogram was normal. After five days, serum calcium level dropped to 10 mg/dL, but the iPTH level increased to 1,374.2 pg/mL (Figures 1, 2). Further evaluation with ultrasonography of the neck revealed two nodular lesions at the right lobe of the thyroid gland. One was a large complex nodule (27 × 24 mm) in the lower pole and the other one was (17 × 14 mm) in the upper pole (Figure 3). Tc99m sestamibi scan showed increased radiotracer uptake in the area of the right thyroid gland suggestive of parathyroid adenoma/carcinoma (Figure 4). The skeletal survey was normal. Renal function tests, thyroid status, serum amylase, creatine kinase, blood sugar, and renal and abdominal imaging revealed no abnormalities. Serum alkaline phosphatase level was slightly increased at 178 U/L (30-120 U/L). Other endocrine screening tests to determine the association with multiple endocrine neoplasias were also done, but all the reports were normal. Additionally, the nerve conduction study was normal. Electromyography showed full recruitment, mixed short and normal duration, and small-amplitude polyphasic potentials in different groups of muscles (quadriceps, biceps brachii, etc.), suggestive of myopathic potential (Figure 5).

We referred the patient to the surgery department for the resection of the nodules. After surgery, two biopsied specimens were sent for histopathology. The larger nodule in the lower pole was diagnosed as PTC, and the smaller nodule in the upper pole was diagnosed as parathyroid adenoma. Post-surgery serum calcium and iPTH levels were 9.0 mg/dL and 41.9 pg/mL, respectively (Figures 1, 2). She was again referred to the surgery department of BSMMU for total thyroidectomy. She was under the care of surgeons and endocrinologists with close monitoring. After one month, she came to the neurology OPD for follow-up. Her weakness was slightly improved; muscle power was grade 4 proximally and grade 5 distally. She was advised to undergo physiotherapy and come for a neurology follow-up after six months. Additionally, she was advised to follow up with the endocrinologists and surgeons to monitor any recurrence of hyperparathyroidism and PCT.

## Discussion

The prevalence of PHP is up to 0.1% in the general population. The usual age of presentation is in the sixth to the seventh decade of life with a female predominance (female-to-male ratio of 3:1). The presentation before 40 years of age is unusual. At an earlier age of onset, the incidence is similar in males and females [3]. Our patient presented at the age of 38 years.

With the increasing use of serum calcium assay in general practice, the most common presentation of PHP has been asymptomatic individuals diagnosed during a routine investigation. Presentation with classical symptoms such as stones, bones, groans, and psychic moans is relatively less nowadays [4]. Variable neuromuscular findings such as proximal weakness, wasting with preserved jerks, or hyperreflexia can be seen. Hyperparathyroidism and hyperthyroidism share this unique pattern of myopathy with hyperreflexia [5]. Moreover, the variable pattern of wasting, including rare distal involvement, may be noted, making hyperparathyroidism a differential diagnosis of motor neuron disease [6,7]. In our patient, marked proximal weakness and wasting were the presenting features. Subsequently, we found a few other features related to the hypercalcemic crisis. For the evaluation of proximal wasting, we performed the nerve conduction study and electromyography, which showed myopathic changes. However, we did not find any other organ involvement such as renal stone, skeletal changes, or pancreatitis. Further, despite the patient’s hypercalcemic crisis, her cognition was remarkably preserved.

The hypercalcemic crisis is a life-threatening medical emergency. The most common cause is malignancy-induced hypercalcemia. Hypercalcemic crisis as a manifestation of PHP is rare. Different case series have shown incidence rates ranging from 1.6% to 6.7% [8]. Unfortunately, the hypercalcemic crisis has no standard definition, an arbitrary definition of an albumin-corrected serum calcium level greater than 14 mg/dL in association with the presence of multiorgan dysfunction has been set up by a few authorities [9]. Our patient initially presented with myopathy and subsequently developed the classical features of hypercalcemic crisis after admission. With aggressive fluid resuscitation, most of her hypercalcemic symptoms and blood calcium level resolved within a few days.

PTC is the most common form of thyroid malignancy. The annual incidence rate ranges from 0.5 to 10 cases per 100,000 individuals depending on the geographical location. The median age of diagnosis is approximately 45 to 50 years, with a female-to-male ratio of about 3:1. Most PTC patients present with asymptomatic thyroid nodules [10].

PTC associated with PHP has been increasingly recognized in recent years. In one case series of PHP patients undergoing surgery, it was found to be associated with concomitant PTC in 2.3-4.3% cases. Most cases had a history of neck irradiation, which might indicate a possible causal relationship [11]. In our patient, we noticed a small non-tender round nodule over the right thyroid lobe, which was thought to be a palpable parathyroid gland. However, eventually, it was diagnosed as concomitant PTC. Some researchers hypothesized that calcium as a carcinogenic agent stimulates angiogenesis and carcinogenesis. Even though this hypothesis is yet to be proven, the increasing association between PHP and PTC has become well-recognized. While researchers will be in search of the causal relationship between these two entities, clinicians must remain vigilant of any suspicious nodule in the thyroid gland during the evaluation of PHP.

## Conclusions

Hyperparathyroidism can present with unusual manifestations such as proximal myopathy and hypercalcemic crisis. Therefore, a high degree of clinical suspicion is needed during the evaluation of myopathy without obvious etiology. The hypercalcemic crisis is a medical emergency. Early diagnosis and treatment are essential in the management of this life-threatening condition. Any thyroid nodule associated with PHP must be evaluated appropriately before surgery to avoid the need for a second operation for possible thyroid malignancy.
